# High USP22 expression indicates poor prognosis in hepatocellular carcinoma

**DOI:** 10.18632/oncotarget.3705

**Published:** 2015-03-30

**Authors:** Bo Tang, Fang Tang, Bo Li, Shengguang Yuan, Qing Xu, Stephen Tomlinson, Junfei Jin, Wei Hu, Songqing He

**Affiliations:** ^1^ Department of Hepatobiliary Surgery, Affiliated Hospital of Guilin Medical University, Guilin, Guangxi, People's Republic of China; ^2^ Guangxi Key Laboratory of Molecular Medicine in Liver Injury and Repair, Guilin, Guangxi, People's Republic of China; ^3^ Department of Pathology, Affiliated Hospital of Guilin Medical University, Guilin, Guangxi, People's Republic of China; ^4^ Department of Microbiology and Immunology, Darby Children's Research Institute, Medical University of South Carolina, Charleston, South Carolina, USA; ^5^ Department of Laboratory Medicine, The First Hospital of Jilin University, Changchun, Jilin, People's Republic of China

**Keywords:** hepatocellular carcinoma, ubiquitin-specific protease 22, prognosis, cancer biomarker

## Abstract

Ubiquitin-specific protease 22 (USP22) removes ubiquitin from histones, thus regulating gene transcription. The expression frequency and expression levels of USP22 were significantly higher in hepatocellular carcinoma (HCC) than in normal liver tissues. High USP22 expression in HCC was significantly correlated with clinical stage and tumor grade. Kaplan-Meier analysis showed that elevated USP22 expression predicted poorer overall survival and recurrence-free survival. High USP22 expression was also associated with shortened survival time in patients at advanced tumor stages and with high grade HCC. Multivariate analyses revealed that USP22 expression is an independent prognostic parameter in HCC. These findings provide evidence that high USP22 expression might be important in tumor progression and serves as an independent molecular marker for poor HCC prognosis. Thus, USP22 overexpression identifies patients at high risk and represents a novel therapeutic molecular target for this tumor.

## INTRODUCTION

Hepatocellular carcinoma (HCC) is the fifth most common malignant tumor worldwide and causes approximately 600,000 deaths globally each year [1]. Despite improvements in clinical treatments such as surgical resection, liver transplantation and interventional therapy, the overall 5-year survival rate of HCC is only 7% [2]. Because tumorigenesis and tumor progression in hepatic cells are caused by multiple genetic alterations, a single molecule targeting therapy has yet to be discovered. Thus, the identification of target molecules that control the biological characteristics of HCC is of great importance.

Ubiquitin-specific protease 22 (USP22) is a recently identified novel human de-ubiquitinating enzyme that is widely expressed in various adult tissues and at the early embryonic stage [3]. Increasing evidence places USP22 at the core of many physiological and pathological processes. USP22 is part of the 11-gene Polycomb/cancer stem cell signature, which uniformly exhibits a marked propensity toward metastatic dissemination and a therapy resistance phenotype [4, 5]. USP22 is also an important subunit of the human Spt-Ada-Gcn5 acetyltransferase (hSAGA) transcriptional cofactor complex, which is required for activator-driven transcription and cell cycle progression [4]. Within the hSAGA complex, USP22 removes ubiquitin from histone H2B, thus regulating the transcription of downstream genes that are related to epigenetic alteration and cancer progression. Moreover, USP22 is required for the proper functioning of MYC, which is widely believed to play a significant role in regulating the tumor cell cycle and tumor invasion [4, 5]. Recent studies have demonstrated that USP22 can inhibit the transcription of the p21 gene by de-ubiquitinating the transcriptional regulator FBP1, leading to cell proliferation and tumorigenesis [6]. We proposed that USP22 might be related to clinical prognosis in several systemic malignancies. In fact, elevated USP22 expression can predict shorter intervals of tumor recurrence, distant metastasis, therapeutic failure and poor prognosis in patients with many types of cancer, including colorectal [7], breast [8], and gastric [9] cancers. However, the clinical significance of USP22 expression in patients with HCC and the role of USP22 in a HCC cell line have not yet been investigated.

The purpose of this study was to clarify the clinical importance of USP22 by analyzing 104 consecutive patients with HCC who had been treated with curative resection and to investigate the functional role of USP22 in HCC cells *in vitro*.

## RESULTS

### USP22 expression in HCC cell lines

**Figure 1:**
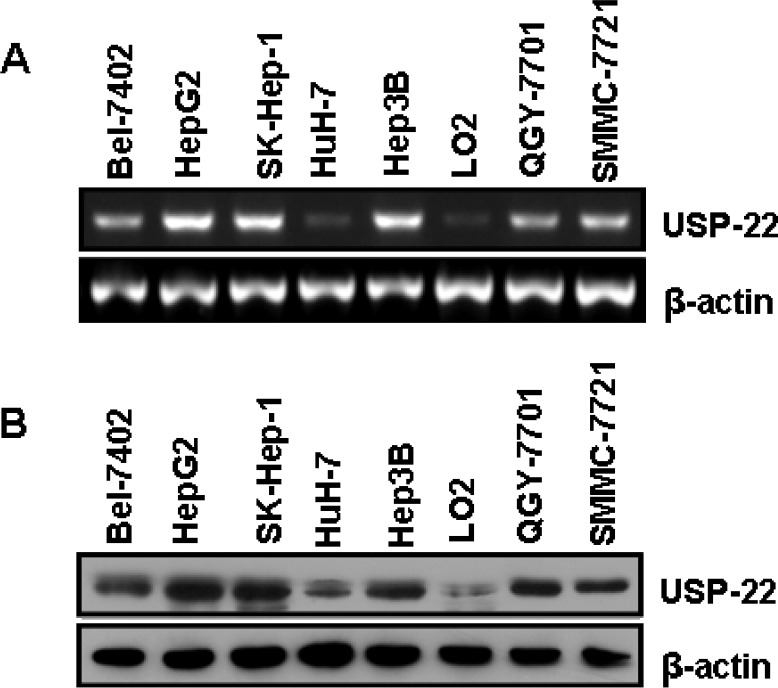
USP22 expression in HCC tissues and cell lines (**A**) Semi-quantitative RT-PCR analysis of USP22 mRNA expression in seven HCC cell lines (Bel-7402, HepG2, SK-Hep-1, HuH-7, Hep3B, QGY-7701, and SMMC-7721) and a normal hepatic cell line (LO2); (**B**) Western blot analysis of USP22 protein expression in seven HCC cell lines (Bel-7402, HepG2, SK-Hep-1, HuH-7, Hep3B, QGY-7701, and SMMC-7721) and a normal hepatic cell line (LO2). β-actin was used as internal control for RT-PCR and Western blot.

USP22 expression was analyzed in seven established human HCC cell lines (Bel-7402, HepG2, HuH-7, SK-Hep-1, Hep3B, QGY-7701 and SMMC-7721) and a normal hepatic cell line LO2 using RT-PCR and Western blot assay. HuH-1 is a well-differentiated HCC cell line, whereas Bel-7402, HepG2, SK-Hep-1, Hep3B, QGY-7701 and SMMC-7721 are moderately to poorly differentiated HCC cell lines. As shown in Fig. 1A and 1B, USP22 expression was significantly higher in the poorly and moderately differentiated cells (Bel-7402, HepG2, SK-Hep-1, Hep3B, QGY-7701 and SMMC-7721) than in the well-differentiated and normal hepatic cells (HuH-1 and LO2). These initial observations revealed that de-ubiquitination might be correlated with HCC differentiation.

### USP22 expression in HCC and normal liver tissues

**Figure 2:**
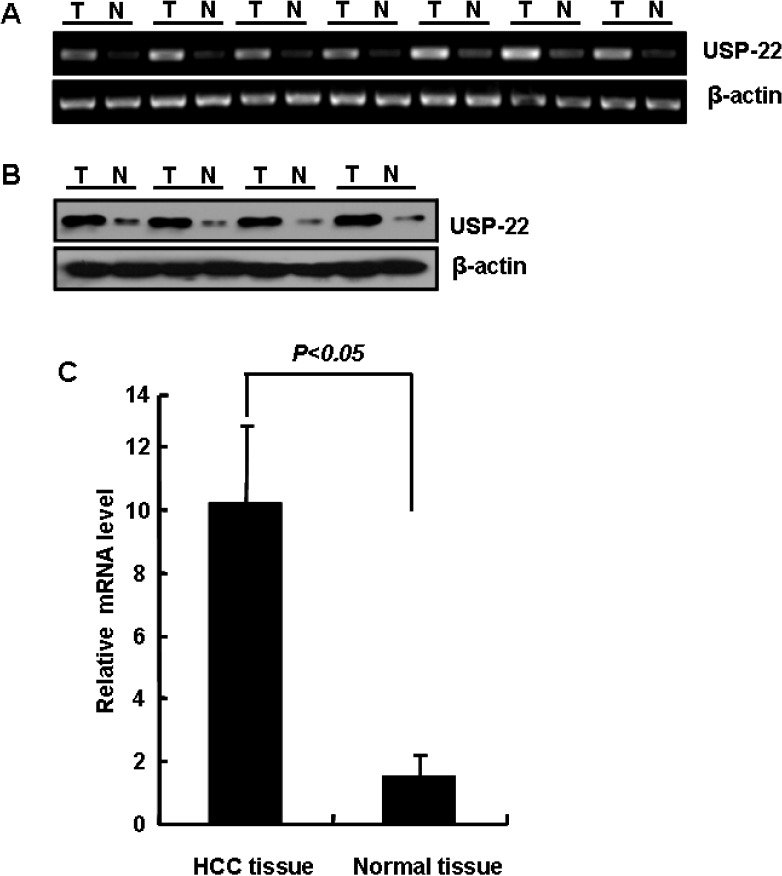
USP22 expression in HCC and normal liver tissues (**A**) Semi-quantitative RT-PCR analysis of USP22 mRNA expression in HCC specimens (T) and normal adjacent hepatic tissue (N); (**B**) Western blot analysis of USP22 protein expression in representative HCC (T) and normal adjacent tissue (N); (**C**) Quantitative RT-PCR analysis of USP22 expression in HCC specimens (T) and normal adjacent hepatic tissue (N). β-actin served as an internal control.

Based on the semi-quantitative RT-PCR analysis of seven pairs of HCC and normal adjacent liver tissues, we found that USP22 mRNA was overexpressed in HCC (Fig. 2A); this finding was confirmed using quantitative RT-PCR analysis (Fig. 2C). To further confirm that USP22 is overexpressed in HCC, the protein levels of USP22 in HCC and matching normal adjacent liver tissues were analyzed using Western blots. As shown in Fig. 2B, the USP22 protein was overexpressed in HCC specimens but not in the matched normal adjacent liver tissues.

### Silencing USP22 expression by siRNA

**Figure 3:**
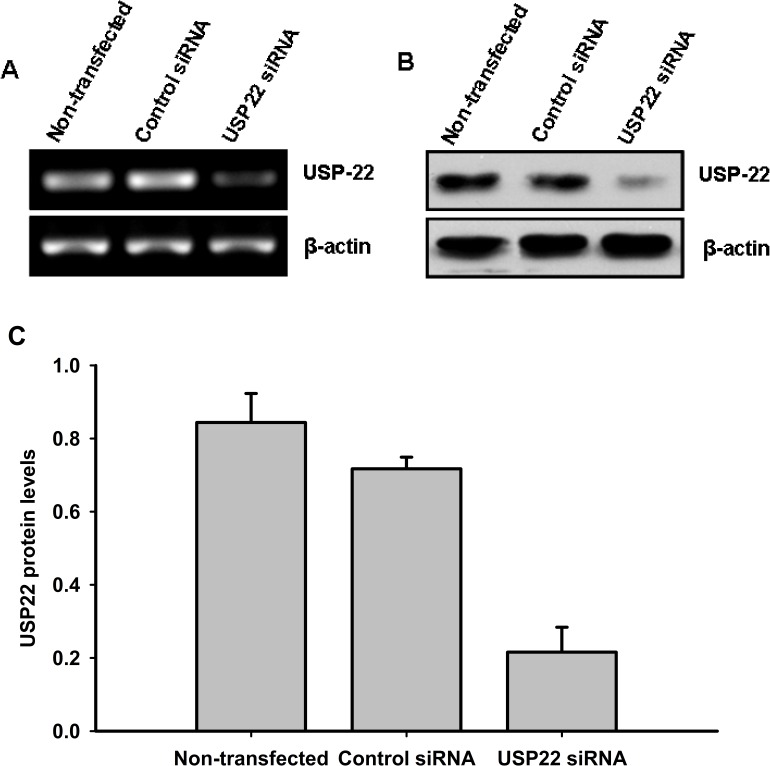
siRNA silencing of USP22 HepG2 cells were transfected with USP22 siRNA and negative control siRNA. At 48 h post-transfection, changes in the mRNA and protein levels of USP22 were determined using RT-PCR (**A**) and Western blot (**B**) analyses. β-actin was used as internal control for Western blot and RT-PCR. (**C**) Relative protein levels were quantified using Image J (NIH, Bethesda, MO, USA) and normalized versus internal control. Three independent experiments were performed and representative results are shown.

RNA interference was performed by transfecting HepG2 cells with control siRNA and USP22-specific siRNA. Following transfection, RT-PCR and Western blot analyses were performed to determine USP22 mRNA and protein expression levels. As shown in Fig. 3, USP22-specific siRNA effectively inhibited USP22 gene transcription and protein expression.

### RNA interference-mediated USP22 gene silencing and growth inhibition of HCC cells

**Figure 4:**
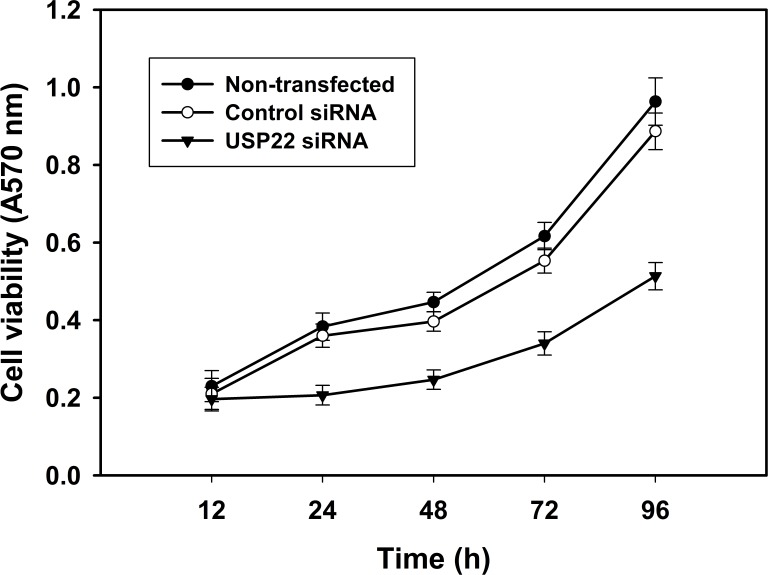
USP22 gene silencing inhibits HCC cell growth HepG2 cells were transfected with USP22 siRNA, negative control siRNA and vehicle control, respectively. At 12, 24, 48, 72 and 96 h post-transfection, cell viability was determined using the MTT assay. Each independent experiment was performed 3 times.

To investigate whether RNA interference-mediated USP22 gene silencing affects the growth of HCC cells, the MTT method was used to analyze HepG2 cell viability at 12, 24, 48, 72 and 96 h following transfection with USP22 siRNA. The results indicate that HepG2 cell viability was significantly lower than in the control groups. These observations indicate that USP22 gene silencing by RNA interference inhibits HCC cell growth (Fig. 4).

### Down-regulation of USP22 expression by siRNA induces the mitochondrial apoptosis of HCC cells

**Figure 5:**
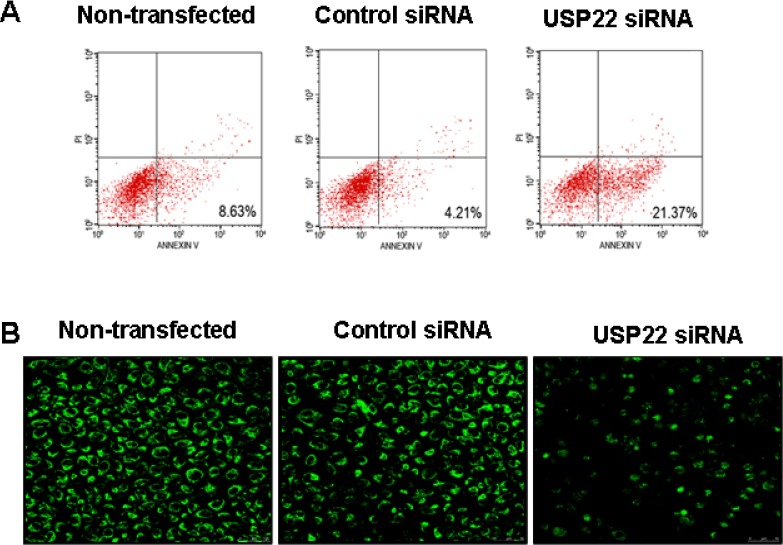
USP22 gene silencing leads to HCC cell apoptosis HepG2 cells were transfected with USP22 siRNA and negative control siRNA. The cells were analyzed at 24 h post-transfection. (**A**) Flow cytometry were performed to analyze cell apoptosis rate. Each independent experiment was performed 3 times. (**B**) USP22 expression was observed in HCC cells via immunohistochemical staining with an anti-USP22 antibody under a confocal laser scanning microscope.

To investigate the mechanism by which USP22 silencing inhibits HCC cells, flow cytometry was used to determine the rate of cell apoptosis, and Western blot analysis was performed to analyze apoptosis-related changes in protein expression levels. Following RNA interference-mediated USP22 gene silencing (24 h), the apoptosis rate of HepG2 cells increased significantly (Fig. 5A). At the same time, we observed changes in the mitochondrial membrane potential using Rhodamine 123 dye. We found that most of the control cells exhibited uniform, bright and clear fluorescence; however, in the USP22-silenced cells, the fluorescence intensity was significantly weaker, stained non-uniformly, and exhibited point-like nucleus fragmentation (Fig. 5B).

**Figure 6:**
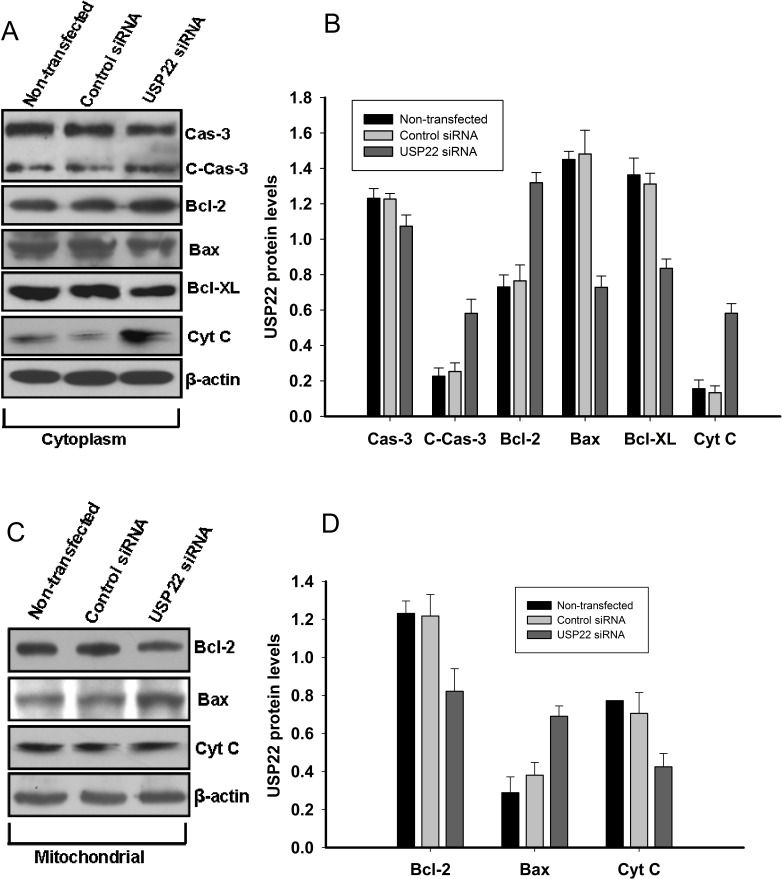
USP22 gene silencing induces mitochondrial apoptosis in HepG2 cells At 48 h post-transfection with USP22 siRNA and control siRNA in HepG2, the mitochondria and protein in the cytoplasm were isolated and subjected to Western blot analysis. β-actin was used as internal control. (**A**). Protein levels of caspase-3(Cas-3), cleaved caspase-3 (C-Cas-3), Bcl-2, Bax, Bcl-XL, and cytochrome c (Cyt C) in the cytoplasm; (**B**) Relative protein expression levels in the cytoplasm were quantified using Image J and normalized versus international control; (**C**). Protein levels of Bcl-2, Bax and CytC in the mitochondria.(**D**) Relative protein expression levels in the mitochondria were quantified using Image J and normalized versus international control density. Three independent experiments were performed and representative results are shown.

We next explored the pathways by which USP22 silencing promoted HepG2 cell apoptosis. Western blot analysis was employed to determine caspase-3, cleaved caspase-3, Bax, Bcl-2, Bcl-XL, and cytochrome C expression in the cytoplasm and mitochondria. Indeed, as shown in Fig. 6, the level of the proapoptotic protein Bax was decreased in the cytoplasm by USP22 silencing, whereas the level of the anti-apoptotic protein Bcl-2 was markedly increased. After USP22 siRNA transfection, the level of cytochrome C protein was decreased in the mitochondria but was increased in the cytoplasm, suggesting the activation of caspase cascades. Furthermore, Western blot analysis confirmed that USP22 silencing had upregulated the cleaved-caspase-3 protein (Fig. 6).

**Figure 7:**
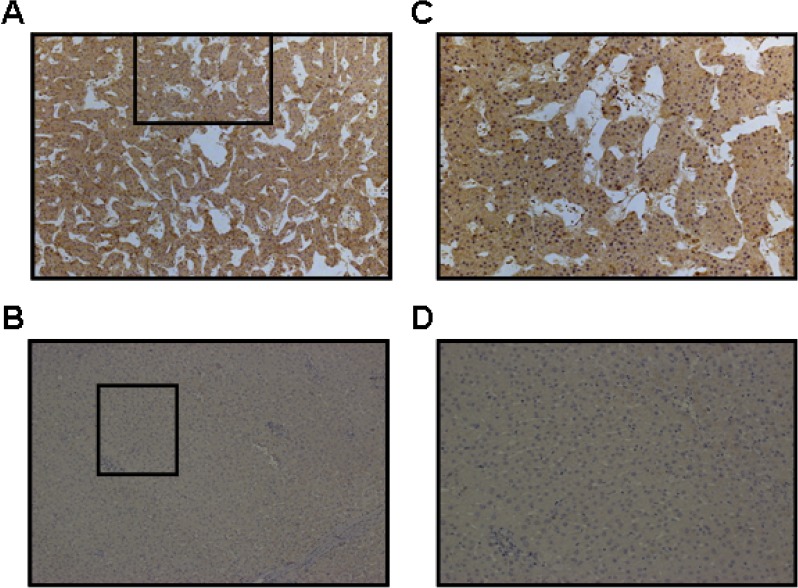
Immunohistochemical staining for USP22 in HCC and normal adjacent hepatic tissues The level of USP22 protein was determined by immunohistochemical staining using a USP22 antibody, and the nuclei were counterstained with hematoxylin. (**A**) USP22 was overexpressed in the cytoplasm in HCC tissue; (**B**) USP22 was expressed at negative levels in matched normal hepatic tissues from the same patient (100×). (**C**) and (**D**) are images recorded at higher magnification (400×) from the areas shown in boxes in (A) and (B), respectively.

**Figure 8:**
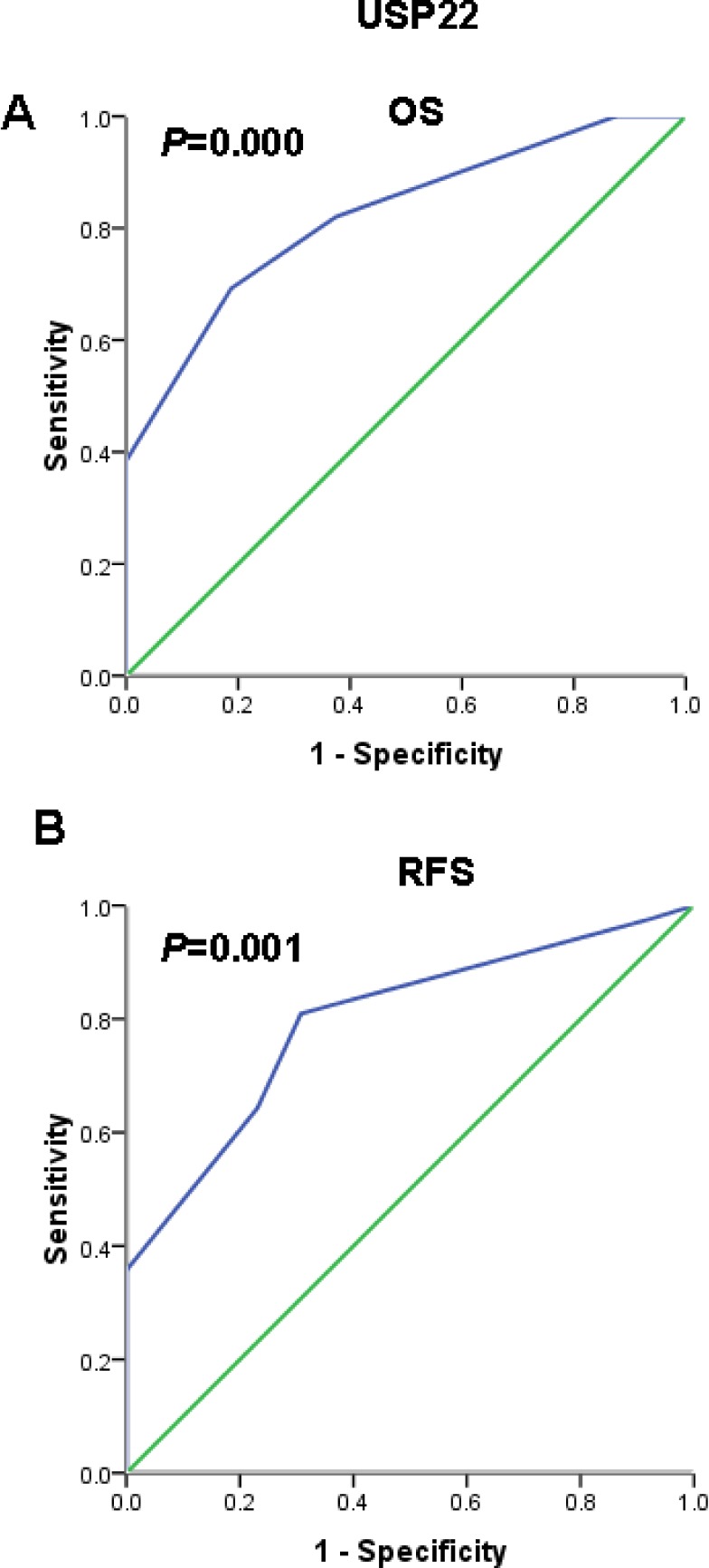
Receiver operating characteristic (ROC) curve analysis used to select a USP22 cutoff score based on the training set (**A**) USP22 cutoff for overall survival in the training set. (**B**) USP22 cutoff for recurrence-free survival in the training set. At each immunohistochemical score, the sensitivity and specificity for the outcome studied were plotted, thus generating a ROC curve.

Immunohistochemical results show that the positive USP22 staining was mainly located in the cytoplasm and was overexpressed in HCC tissue, whereas normal matched liver tissues showed negative expression (Fig. 7). To further assess survival and avoid the problems of multiple cut point selection, ROC curve analysis was employed to determine a cutoff score for USP22 expression. As shown in Figs. 8A and 8B, the USP22 cutoff score for OS and PFS in the training set was 3.5 (*p* = 0.000 and *p* = 0.001, respectively). We thus selected a USP22 expression score of 3.5 (>3.5 VS.≤ 3.5) as the uniform cutoff point for survival analysis in the test set.

Taken together, these findings strongly indicate that USP22 silencing triggered the mitochondrial apoptosis pathway that is associated with caspase 3 activation in HCC cells.

### USP22 expression and clinical features

The clinical features of the two studied patient cohorts, including age, gender, clinical stage, tumor size, tumor number, tumor grade, serum AFP level and USP22 expression, are summarized in Table 1. The ROC-derived USP22 cutoff score of 3.5 developed from the training set successfully segregated the test set into high (34/59, 57.6%) and low (25/59, 42.4%) USP22 expression subgroups. High USP22 expression was mainly found in patients with more advanced tumor stages (50/74 in stages III+IV vs. 8/30 in stages I+II, *p* = 0.000) and high-grade tumors (23/24 in grades 3-4 vs. 35/80 in grades 1-2, *p* = 0.000). Furthermore, correlation analysis demonstrated that high USP22 expression was correlated with clinical stage (*p*= 0.002 for the training set and *p*= 0.026 for the test set) and tumor grade (*p*= 0.004 for the training set and *p* = 0.001 for the test set). USP22 associated with patient age in the training set (*p* = 0.01) but not in the test set. We failed to detect any relationship between USP22 and other patient characteristics, including age, gender, tumor number and serum AFP level.

**Table 1:** Association of USP22 expression with patient's characteristics in hepatocellular carcinoma

Variable	All cases	Training set (n = 45)			Testing set (n = 59)		
		High expression	Low expression	p^a^	High expression	Low expression	p^a^
Age (years)				0.259			0.6481
≥50.00 ^b^	57	12(26.7%)	14(31.1%)		17(28.8%)	14(23.7%)	
<50.00	47	12(26.7%)	7(15.5%)		17(28.8%)	11(18.6%)	
Gender				1			0.061
Male	94	23(51.1%)	20(44.4%)		32(54.2%)	19(32.2%)	
Female	10	1(0.2%)	1(0.2%)		2(3.4%)	6(10.2%)	
Clinical stage				0.002			0.026
I+II	30	4(8.9%)	13(28.9%)		4(6.8%)	9(15.3%)	
III+IV	74	20(44.4%)	8(17.8%)		30(50.8%)	16(27.1%)	
Tumor size (cm)				0.003			0.829
≤5	48	9(20.0%)	17(37.8%)		9(15.3%)	6(10.2%)	
>5	56	15(33.3%)	4(8.9%)		25(42.3%)	19(32.2%)	
AFP (ng/mL)				0.202			0.113
<200	57	19(42.2%)	13(28.9%)		29(49.2%)	17(28.8%)	
≥200	47	5(11.1%)	8(17.8%)		5(8.5%)	8(13.5%)	
Edmonson grade				0.004			0.001
1-2	80	16(35.5%)	21(46.7%)		19(32.2%)	24(40.7%)	
3-4	24	8(17.8%)	0(0%)		15(25.4%)	1(1.7%)	
Tumor number				0.259			0.589
solitary	59	12(26.7%)	14(31.1%)		18(30.5%)	15(25.4%)	
mutiple	45	12(26.7%)	7(15.5%)		16(27.2%)	10(16.9%)	

a x^2^ test.

b median age

### USP22 expression and survival analysis: univariate survival analysis

**Figure 9:**
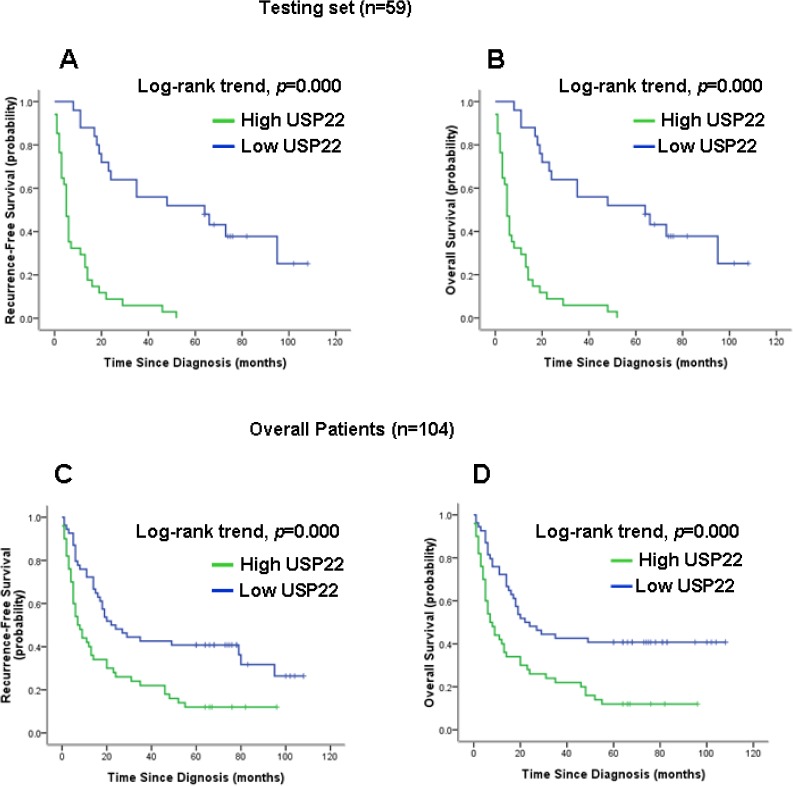
Kaplan-Meier survival analysis of USP22 expression in the test set and in all patients Higher USP22 expression was closely correlated with (**A**) poor overall survival and (**B**) recurrence-free survival in the test set. (**C**) Higher USP22 expression also correlated with inferior overall survival (**D**) and recurrence-free survival in all patients. In the test set and in all patients, the median durations of overall survival for patients with low and high USP22 expression were 78.0 vs. 15.0 months (*p* < 0.001) and 82.0 vs. 22.0 months (*p* < 0.001), respectively.

Kaplan-Meier analysis (Figs. 9B and 9D) showed that elevated USP22 expression strongly predicted inferior OS in the test set (*p*< 0.001) and overall (*p*< 0.001). Moreover, USP22 expression was a powerful prognostic factor for RFS in the test set (*p*< 0.001, Fig. 9A) and overall (*p*< 0.001, Fig. 9C).

### Multivariate Cox regression analysis

To avoid the effects of univariate analysis, USP22 expression and other parameters were examined using multivariate Cox analysis (Tables 2 and 3). In the test set, USP22 was indeed found to be a significant independent prognostic factor for poor OS (hazard ratio, 4.973; 95% CI, 2.115-11.693; *p* < 0.001; Table 2) and RFS (hazard ratio, 4.943; 95% CI, 2.100-11.636; *p* < 0.001; Table 2). Similar results were also observed for all patients (hazard ratio, 4.981; 95% CI, 2.630-9.434; *p* < 0.001 for OS, and hazard ratio, 4.979; 95% CI, 2.629-9.426; *p* < 0.001 for RFS; Table 3). Clinical stage and tumor grade were found to be independent prognostic factors for patient survival in the test set and for all patients.

**Table 2:** Results of multivariate Cox proportional-hazards analysis in testing set

Variable	For death			For recurrence-free survival		
	Hazard Ratio	95% confidence interval	*p*	Hazard Ratio	95% confidence interval	*p*
Age <50.00 years (VS. ≥50years)	0.558	0.297 to 1.050	0.07	0.558	0.297 to 1.047	0.069
Gender male (VS. female)	0.774	0.263 to 2.276	0.642	0.752	0.255 to 2.215	0.605
Clinical stage IV+III (VS. II+I)	3.467	1.197 to 10.037	0.022	3.289	1.136 to 9.527	0.028
Tumor size ≤5.0cm	1.366	0.656 to 2.843	0.404	1.339	0.646 to 2.777	0.432
(VS.>5.0cm)						
AFP <200ng/mL (VS. ≥200ng/mL)	1.149	0.514 to 2.573	0.735	1.127	0.504 to 2.520	0.771
Edmonson grade	5.075	2.052 to 12.551	0	5.001	2.013 to 12.424	0.001
Low (vs.high)						
Tumor number solitary (vs. mutiple)	1.072	0.539 to 2.134	0.842	10.6	0.534 to 2.106	0.867
USP22 Positive (vs. negative)	4.973	2.115 to 11.693	0	4.943	2.100 to 11.636	0

**Table 3:** Results of multivariate Cox proportional-hazards analysis in overall patients

Variable	For death			For recurrence-free survival		
	Hazard Ratio	95% confidence interval	*p*	Hazard Ratio	95% confidence interval	p
Age <50.00 years (VS. ≥50years)	0.55	0.347 to 0.872	0.011	0.549	0.347 to 0.870	0.011
Gender male (VS. female)	1.056	0.430 to 2.596	0.905	0.549	0.347 to 0.870	0.011
Clinical stage IV+III (VS. II+I)	3.537	1.626 to 7.692	0.001	1.044	0.425 to 2.564	0.926
Tumor size ≤5.0cm	1.403	0.819 to 2.401	0.217	3.501	1.608 to7.623	0.002
(VS.>5.0cm)						
AFP <200ng/mL (VS. ≥200ng/mL)	1.18	0.643 to 2.165	0.593	1.379	0.807 to 2.356	0.24
Edmonson grade	3.854	1.998 to 7.437	0	1.18	0.645 to 2.161	0.591
Low (vs.high)						
Tumor number solitary (vs. mutiple)	0.901	0.531 to 1.529	0.699	3.847	1.992 to 7.431	0
USP22 Positive (vs. negative)	4.981	2.630 to 9.434	0	0.892	0.526 to 1.514	0.673

## DISCUSSION

HCC is one of the most common cancers worldwide and poses a serious public health problem [1]. Diagnosis at an advanced stage and high resistance to conventional systemic therapy remain the main causes for the poor survival of HCC patients [10]. Although previous studies have found that many aberrantly expressed genes in liver tumors can help to predict patient risk [11-14], additional novel molecular markers that can identify tumor progression and predict individual prognosis are urgently needed. USP22 has recently been identified as a novel human de-ubiquitinating enzyme. Elevated USP22 expression can predict shorter interval of tumor recurrence, distant metastasis, therapeutic failure and poor prognosis in patients with many cancer types [15-18]. However, the expression dynamics and biological role of USP22 in HCC remain unclear. In the present study, we detected USP22 expression in HCC cell lines and cancer tissues. Similar to the findings of previous studies [16, 18], USP22 was found to be expressed at higher levels in poorly differentiated cancer cell lines and cancer tissues and to correlate closely with HCC differentiation (Fig. 1). To explore the biological function of USP22 in HCC cells, USP22-specific siRNA was transfected into HepG2 cells. The effects of USP22 silencing on cell proliferation and apoptosis were investigated in HepG2 cells. Our results demonstrated that USP22 silencing suppressed cell growth (Fig. 4) and induced cell apoptosis (Fig. 5). Further analyses showed that USP22 silencing in HepG2 cells decreased the Bcl-2/Bax ratio and enhanced the release of cytochrome c into the cytoplasm (Fig. 6), suggesting the initiation of mitochondrial-mediated apoptosis. In addition, caspase-3 was activated (Fig. 6), indicating that caspase-associated apoptosis was induced by USP22 silencing. Collectively, our results show that USP22 silencing in HepG2 cells suppressed cell growth through mitochondrial apoptosis and that this suppression was dependent on caspase activation.

Next, to develop an objective USP22 cutoff for survival analysis, we used ROC curve analysis to generate a cutoff score using the training set. USP22 expression, which was classified as high or low based on the ROC-derived cutoff, was mainly found to be higher in patients with more advanced tumor stages (stages III and IV) and higher tumor grades (grades 3-4), indicating that USP22 might be involved in HCC progression. Correlation analysis further demonstrated that high USP22 expression was associated with clinical stage and tumor grade in HCC (Table 1). Furthermore, in the test set and in all patients, high USP22 expression predicted a significant OS and RFS disadvantage over the low USP22 expression subgroup (Fig. 8). In addition, multivariate analyses for the test set and for all patients revealed that USP22 expression represents an independent prognostic parameter.

The results indicated that high USP22 expression promotes tumor progression in HCC. USP22 might de-ubiquitinate H2A and H2B, subunits of the human SAGA complex that are intimately linked to the transcriptional activation of the MYC gene and increased cell proliferation in HCC [19]. The activation of the BMI-1 oncogene-associated PcG pathway is known to be a common oncogenic event and a key regulatory mechanism of the “stemness” function in both normal and cancer stem cells. As a novel de-ubiquitinating enzyme with ubiquitin hydrolase activity, USP22 might inhibit apoptosis in HCC by activating the BMI-1-mediated PcG stem cell pathway [20].

In summary, this study demonstrates that USP22 expression is significantly increased in HCC tissues and that the increased USP22 expression in human HCC might be important for tumor progression and can serve as an independent biomarker for poor survival. Further analysis demonstrated that USP22 downregulation activated mitochondrial apoptosis by regulating several apoptosis-related proteins, including Bcl-2, Bax, cytochrome c and caspase-3. Thus, USP22 overexpression in HCC identifies patients at high risk of progression and recurrence and is a novel therapeutic molecular target for HCC.

## MATERIALS AND METHODS

### Ethics statement

All experimental procedures were undertaken at the Affiliated Hospital of Guilin Medical University. The study was approved by the Ethics Committee of Guilin Medical University. Written informed consent was received from all patients, and the ethical guidelines described in the Declaration of Helsinki were followed.

### Cell culture and siRNA transfection

HCC cell lines including HepG2, Bel-7402, SK-Hep-1, HuH-7, Hep3B, QGY-7701, SMMC-7721 and a human normal hepatic cell line, LO2, were purchased from the American Type Culture Collection (ATCC). Cells were cultured in Iscove's modified Dulbecco's medium (IMDM) containing 10% fetal bovine serum (FBS) and maintained at 37°C under 5% CO_2_. USP22 sequence-specific siRNA and negative control siRNA (Guangzhou Ribobio Co., Ltd.) were designed and synthesized as described previously [21].

### RNA isolation and gene expression using semi-quantitative RT-PCR and quantitative real time RT-PCR

Total RNA from hepatic tissue and cell lines was prepared using RNAiso^TM^Plus (Takara) according to the recommended method. RNAs were reverse transcribed using the PrimeScript 1st Strand cDNA Synthesis Kit (TAKARA) according to the manufacturer's instructions. The real-time reverse transcription-polymerase chain reaction (RT-PCR) was performed using an RT-PCR kit according to the protocols recommended by the manufacturer. A SYBR green-based RT-PCR assay was used to determine the mRNA level of USP22 in HCC and matched normal adjacent liver tissues using the ABI PRISM 7900HT Sequence Detection System (Applied Biosystems, Foster City, CA). β-actin was used as an internal control to normalize gene expression levels. The primers used for amplification were as follows: β-actin, forward primer, 5′-AAGGAAGGCTGGAAGAGTGC-3′, reverse primer, 5′-CTGGGACGACATGGAGAAAA-3′; USP22, forward primer, 5′-GGCGGAAGATCACCACGTAT-3′, reverse primer, 5′-TTGTTGAGACTGTCCGTGGG-3′.

### Western blot analysis

The hepatic tissue and cell lines were ground and lysed in RIPA buffer on ice and then subjected to Western blot analysis. The mitochondrial and cytosolic fractions were separated using differential centrifugation as described previously [22]. Protein concentrations were determined using the Bradford method, and BSA (Sigma-Aldrich) was used as the standard. Equal amounts of cell and tissue extract (40μg) were subjected to SDS-PAGE, and the resulting bands were transferred to a nitrocellulose membrane (Bio-Rad). The membrane was blocked and then incubated with primary and secondary antibodies. The primary antibodies used were raised against USP22 (abcam, ab71732), Bcl-2, Bcl-XL, Bax, Bad, cytochrome C, caspase-3 and β-actin (ZSGB-BIO, TA-09).

### Cell viability

Cell viability was determined using the MTT method. Cells (2×10^4^ cell/well) were cultured in 96-well culture plates 1 day prior to siRNA transfection. On day 2, after the cells had adhered to the culture plates, the cells were transfected with USP22 and control siRNA. MTT solution (5 mg/ml; 20 μl) was added to each well, and the cells were then cultured in an incubator containing CO_2_ for 4 h. The culture solution was then removed, and 150 μl DMSO was added to each well; the plate was then agitated at room temperature for 10 min. The OD values of each well were measured using a microplate reader. Each experimental group was analyzed in six double wells. The average values were calculated and used to construct growth curves.

### Test of apoptosis using flow cytometry

Cells from all experimental groups were digested in 0.25% trypsin and resuspended in PBS to prepare single cell suspensions. Cell density was adjusted to 1×10^6^ cell/ml. Next, 5 μl Annexin V-FITC and 5 μl PI were added, and the cells were then incubated for 30 min at 4°C prior to flow cytometry analysis.

### Measurement of the mitochondrial membrane protential (MMP)

Mitochondrial membrane potential was determined using Rhodamine 123, a cationic fluorescent indicator that selectively accumulates within mitochondria in a membrane potential-dependent manner. Cells (1×10^6^ cell/ml) were incubated with Rhodamine 123 (100 μg/L) for 45 min at 37°C. Rhodamine 123 fluorescence was measured over the entire field of view under a fluorescent microscope that was connected to an imaging system (BX50-FLA, Olympus, Tokyo, Japan). In addition, 10^4^ cells per sample were analyzed using flow cytometry (FCM, Beckman-Coulter Co., USA); the mean fluorescent intensity (MFI) in the positive cells represents the MMP level.

### Patients

A total of 168 primary HCC patients from the archives of the Department of Pathology at the Affiliated Hospital of Guilin Medical University (Guilin, China) were initially recruited in our study. All patients underwent complete surgical resection from March 2002 to January 2007. We further screened patients according to a strict eligibility criteria protocol as follows: microscopically confirmed hepatocellular carcinoma; without any metastatic diseases; no prior chemotherapy or transhepatic arterial embolization history; having a follow-up period of over 5 years. Ultimately, 52 patients with loss of follow-up and 12 patients who were deficient in clinical characteristics were excluded from this study; 104 HCC patients were subjected to further clinical and survival analysis. The overall cohort comprised 94 male and 10 female patients with a median age of 50 years (range, 22 - 78 years). Eighty-five patients were censused as dead during the 5 years of follow-up, including 2 patients who died from postoperative complications and 83 who died as the result of tumor progression. Of all patients, 45 were randomly assigned by computer (SPSS 16.0 software) to the training set, and the remaining 59 patients were assigned to the test set. The clinicopathological variables of the two cohorts, including age, gender, clinical stage, tumor size, tumor number, serum alpha-fetoprotein (AFP) level and tumor grade, were included in this study. All tumors were classified and staged according to American Joint Commission on Cancer system. Tumor grade was based on the criteria proposed by Edmonson and Steiner. We obtained prior patient consent and approval from the Institute Research Ethics Committee of Guilin Medical University for the use of the clinical materials described in the present study.

### Immunohistochemical analysis and evaluation

Slides (4 μm) were deparaffinized in xylene, rehydrated in graded alcohol, immersed in 3% hydrogen peroxide for 10 min to block endogenous peroxidase activity, and antigen retrieved by pressure cooking for 3 min in Tris/EDTA (pH = 8.0). Then, the slides were incubated with a primary USP22 antibody (monoclonal goat; 1:50; abcam, ab71732) for 1 hour at room temperature. After incubation with a secondary antibody for 30 min, the specimens were stained with DAB (3, 3-diaminobenzidine). Finally, the sections were counterstained with hematoxylin, dehydrated and mounted. A negative control was obtained by replacing the primary antibody with a normal murine IgG. Known immunostaining-positive esophageal squamous cell carcinoma slides were used as positive controls, as previously described [23].

Brown granules in the cytoplasm of USP22 were considered positive staining. We scored the staining intensity as follows: 0, no staining; 1+, mild staining; 2+, moderate staining; 3+, intense staining. The area of staining was evaluated as follows: 0, no staining of cells in any microscopic field; 1+, < 30% of tissue stained positive; 2+, between 30% and 60% of tissue stained positive; 3+, > 60% of tissue stained positive. USP22 expression was evaluated by combined the assessing of staining intensity and extension. The minimum score when summed (intensity+ extension) was 0, and the maximum score was 6. The criteria used in this study are widely accepted [24]. USP22 expression was assessed and scored by two independent pathologists (Drs. F Tang and WF Mo), who were blinded to the clinicopathological data. The agreement between these two pathologists on the IHC score reached 87% (90 identical scores out of 104 cases), suggesting that our scoring system was highly reproducible. If the results reported by the two pathologists were consistent, the value was used in the study. However, interobserver disagreements (approximately 6% of all cases) were reviewed, and a conclusive judgment was agreed by both pathologists.

### Selection of a cutoff score for USP22-positive expression

The selection of a USP22 cutoff score for the training set was based on ROC curve analysis, as described previously [25]. Briefly, the sensitivity and specificity of the studied outcome at each score was plotted to generate a ROC curve. The score closest to the point at which sensitivity and specificity were maximal (0.0, 1.0) was selected as the cutoff score; using this cutoff, the greatest number of tumors was correctly classified as having or not having the outcome. To facilitate ROC curve analysis, the survival features were dichotomized: survival (death vs. others (censored, alive or dead from other causes)).

### Follow up

All patients had follow-up records that were longer than 5 years. After the completion of therapy, patients were observed at 3-month intervals during the first 3 years and at 6-month intervals thereafter. Overall survival was defined as the time from diagnosis to the date of death or, if patients were still alive, when censused at the latest date. Recurrence-free survival was defined as the time from diagnosis to the date of recurrence or, when censused at the latest date, the date of death.

### Statistical analysis

Values are presented as the means ± SD. Statistical analysis was performed using Student's t-test. For survival analysis, the optimal cutoff for USP22 expression was obtained using ROC analysis of the training set (n = 45). For validation, the relationships between USP22 expression (which was classified according to the ROC analysis-generated cutoff point) and either OS or RFS were evaluated for the test set (n = 59) and all patients (n = 104). The chi-square test or Fisher's exact test was employed to evaluate the relationship between USP22 expression and the studied clinicopathological variables. The multivariate Cox proportional hazards model was used to estimate hazard ratios and 95% confidence intervals for patient outcome. The relationships between USP22 expression and either OS or RFS were determined using a Kaplan-Meier analysis. Log-rank tests were performed to measure the differences in survival probabilities between patient subsets. All p values quoted were two-sided, and *p* values < 0.05 were considered statistically significant. Statistical analysis was performed using SPSS v. 16.0 (SPSS, Inc., Chicago)

## Abbreviations

HCC: Hepatocellular carcinoma
USP22: Ubiquitin-specific protease 22
MMP: mitochondrial membrane potential
CLSM: Confocal Laser Scanning Microscopic
